# Extended Tibial Tuberosity Osteotomy: A Practical Tool for Implant Removal in Difficult Knee Revision Arthroplasties in Patients with Hemophilia

**DOI:** 10.3390/medicina61091670

**Published:** 2025-09-15

**Authors:** Dimitrios Kalatzis, Georgios Zoumpoulis, Konstantinos Zygogiannis, Konstantinos Kaoullas, Ioannis Fotoniatas, Anna Kouramba, Georgios Thivaios

**Affiliations:** 1 Trauma and Orthopaedics Department, Laiko General Hospital of Athens, 11257 Athens, Greece; gzoumpoulis@yahoo.gr (G.Z.); zygogianniskonstantinos@gmail.com (K.Z.); ckaoullas28@gmail.com (K.K.); fotoniatas@yahoo.com (I.F.); thevaeos@yahoo.gr (G.T.); 2 Blood Transfusion Service and National Reference Center for Congenital Bleeding Disorders, ‘’Laiko’’ General Hospital, 11527 Athens, Greece; akouraba@otenet.gr

**Keywords:** extended tibial tuberosity osteotomy, hemophilic arthropathy, revision total knee arthroplasty, tibial stem removal

## Abstract

*Background and Objectives*: Hemophilic arthropathy, the end result of recurrent hemarthroses in patients with hemophilia, often necessitates total knee arthroplasty (TKA) using constrained implants to address severe deformities and joint destruction. Revision TKA is often required due to aseptic loosening, implant malposition, infection, or periprosthetic fractures. The extended tibial tuberosity osteotomy (ETTO) has emerged as a critical technique for the safe removal of well-fixed tibial stems in such complex cases, demonstrating high union rates and minimal complications. The aim of this study is to evaluate the safety, effectiveness, and clinical outcomes of the ETTO technique during complex revision TKA in patients with hemophilia. *Materials and Methods*: A retrospective analysis was conducted on seven male hemophilic patients who underwent revision TKA with ETTO between 2015 and 2023. The procedure involved the creation of an extended proximal tibial bone flap, laterally retracted to facilitate tibial stem exposure and removal. Postoperative outcomes included radiological confirmation of osteotomy union, assessment of complications, and evaluation of functional outcomes, including range of motion and extensor mechanism integrity. *Results*: Osteotomy union was achieved in all patients (mean age 57.5 ± 1.50 years and mean body mass index 26.07 ± 0.67 kg/m^2^) within four months, confirmed by radiographic evidence of bridging callus. No significant complications, such as nonunion, fragment displacement, or symptomatic hardware, were observed. There was one patient who experienced delayed wound healing, managed successfully with surgical debridement. Postoperative mean knee flexion was 92°, with no extensor lag reported. ETTO enabled safe tibial stem removal and successful revision arthroplasties in all cases. *Conclusions*: ETTO is a technically demanding but indispensable approach for addressing the challenges of revision TKA in patients with hemophilia. It allows for secure tibial stem removal while maintaining excellent union outcomes and a low rate of complications. Due to its complexity, ETTO should be performed by experienced surgeons in specialized centers.

## 1. Introduction

Hemophilia A and B are inherited X-linked coagulation disorders caused by deficiency of factor VIII (FVIII) and factor IX (FIX), respectively. Hemophilia A is more prevalent (80% to 85% of the total hemophilia population) than hemophilia B. It presents in 1 in 5000 live male births, whereas hemophilia B presents in 1 in 30,000 live male births. Patients exhibit a range of clinical manifestations depending on the factor levels. Severe disease is defined as <1 % factor activity, whereas 1–5 and >5% account for moderate and mild disease, respectively. Men within a family demonstrate the same level of deficiency since they share the same genetic defect. The most common bleeding sites, accounting for approximately 80% of total bleeding episodes, involve the target joints, i.e., ankles, knees and elbows. The settings in which hemorrhage occurs vary according to the severity of the disease. Thus, spontaneous hemarthroses occur at an early age in cases of severe disease, while patients with mild hemophilia display bleeding in joints only following trauma/injury or surgery [1].

Multiple factors contribute to chronic synovitis and joint destruction in hemophilic patients. The synovium has a limited capacity when it comes to blood absorption [2]. Recurrent hemarthroses lead to hemosiderin and ferritin accumulation, intracellular deposition of blood-breakdown products in synovial macrophages, and synovial membrane hypertrophy and neo-angiogenesis. The now hypertrophic direct effect of blood and iron on chondrocytes causes their apoptosis and cartilage damage [3]. Recurrent bleeding episodes eventually cause hemophilic arthropathy. Primary prophylactic treatment starting early in life can reduce bleeding occurrence and the risk of subsequent arthropathy [1]. However, due to lack of patient compliance and the late introduction of factors in use, many patients have ended up with end-stage hemophilic arthropathy.

Hemophilic arthropathy typically requires a more constrained type of implant when a primary total knee arthroplasty (TKA) is performed [4,5]. A constrained prosthesis is necessary mainly due to (a) the severe axial deformity combined with presence of large bone defects and (b) the limited range of motion (ROM) of the hemophilic knee. We acknowledge the need to revise such a constrained TKA in hemophilic patients when we deal with situations like aseptic loosening, mechanical derangement, infection, periprosthetic fracture, or correction of residual deformity caused by malposition of implants. In the latter case, the extraction of well-fixed (cemented or uncemented) long stems can be quite challenging. We therefore use a new technique to safely remove a well-fixed tibial component with a long stem, to avoid catastrophic complications such as a perioperative fracture that could severely reduce the remaining bone stock. In our hands, extended tibial tuberosity osteotomy (ETTO) has been proven to be a necessary tool in these situations in order to safely extract the tibial stem, demonstrating high union and low complication rates [6]. ETTO involves the creation of a large proximal tibial bone flap, retracted laterally to allow direct exposure into the intramedullary canal and long tibial stem removal. It is a complex yet efficient procedure that should only be performed at a referral center.

The purpose of this study is to evaluate the safety, effectiveness, and clinical outcomes of the ETTO technique during complex revision TKA in patients with hemophilia. These patients present a unique surgical challenge due to poor bone quality, soft tissue contractures, and increased bleeding risk.

## 2. Materials and Methods

Pre-operative assessment included thorough clinical examination, long standing x-rays of the affected limb, anteroposterior and lateral views of the knee, tibia, and femur (evaluation of stem length, position, and bone cement presence), and skyline Merchant view in order to assess for patellofemoral arthritis and patella tracking. A routine aspiration of the knee joint and serum ESR and CRP values were always obtained to rule out septic prosthetic joint loosening.

Coagulation factors were administered 30 min preoperatively, and their levels were monitored regularly throughout the surgery. A tourniquet was inflated at a pressure of 300 mm Hg. We performed a midline standard incision in line with the previous scar with a distal extension to allow tibial tubercle exposure. After the creation of two subcutaneous full-thickness flaps (one medial and one lateral), we proceeded with a medial parapatellar arthrotomy and a meticulous release of the suprapatellar pouch, the medial and lateral gutter, and the medial edge of the tibia. If knee flexion was impossible, we routinely performed a quadriceps snip technique to improve knee exposure.

An extended osteotomy of the tibia was performed, using an oscillating saw with a fine blade (0.4 mm thick). Special care was given to minimize the heat produced by saturation with normal saline. Two parallel cuts to the tibial shaft were made on either side of the tibial tubercle at a distance of approximately 5 cm. These cuts were united distally with a transverse cut. The lateral soft tissues were retained as far as possible to maximize vascularization of the osteotomy.

**Figure 1:**
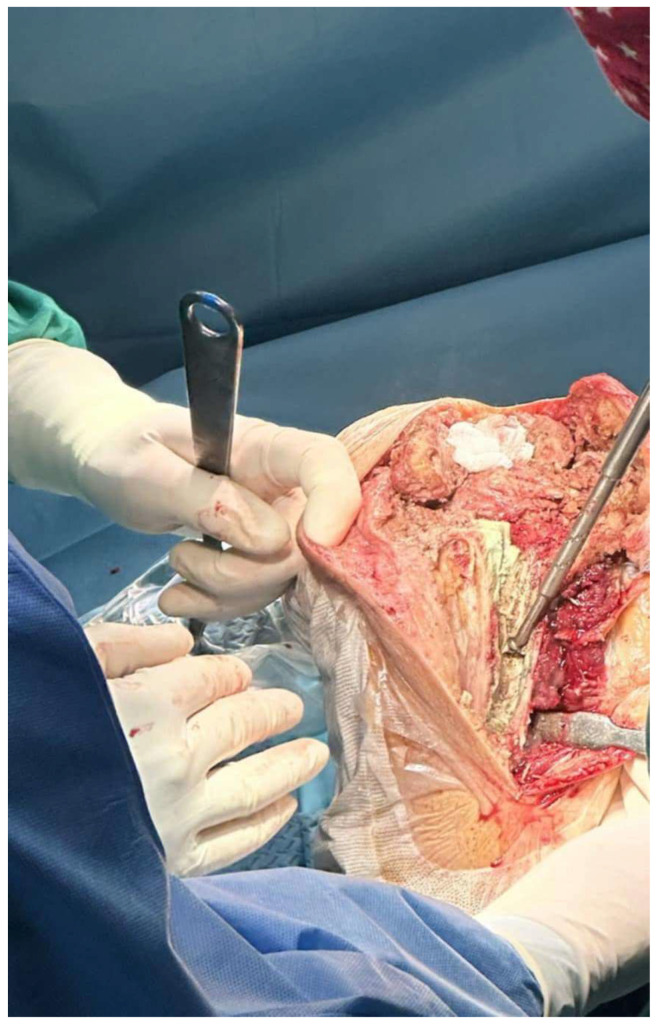
Removal of bone cement from intramedullary canal after the extraction of tibial stem with the use of an ultrasound probe (Case 3).

The length of the osteotomy was decided according to the preoperative radiographs and the length of the implant, entirely revealing the tibial stem. The mean length of the osteotomy fragment in our series was approximately 11 cm. The osteotomy was initially marked with the use of a ruler and an electrocautery. The depth of the osteotomy was aimed to be as deep as possible, but while performing ETTO in revision cases with tibial stems, it was limited by the presence of the tibial stem. The stem was then mobilized using osteotomes and pneumatic drills throughout its circumference and was extracted by hitting the baseplate cephalically. After the stem extraction, the intramedullary remaining cement was meticulously removed, usually with the aid of an ultrasound device (Figure 1).

**Figure 2:**
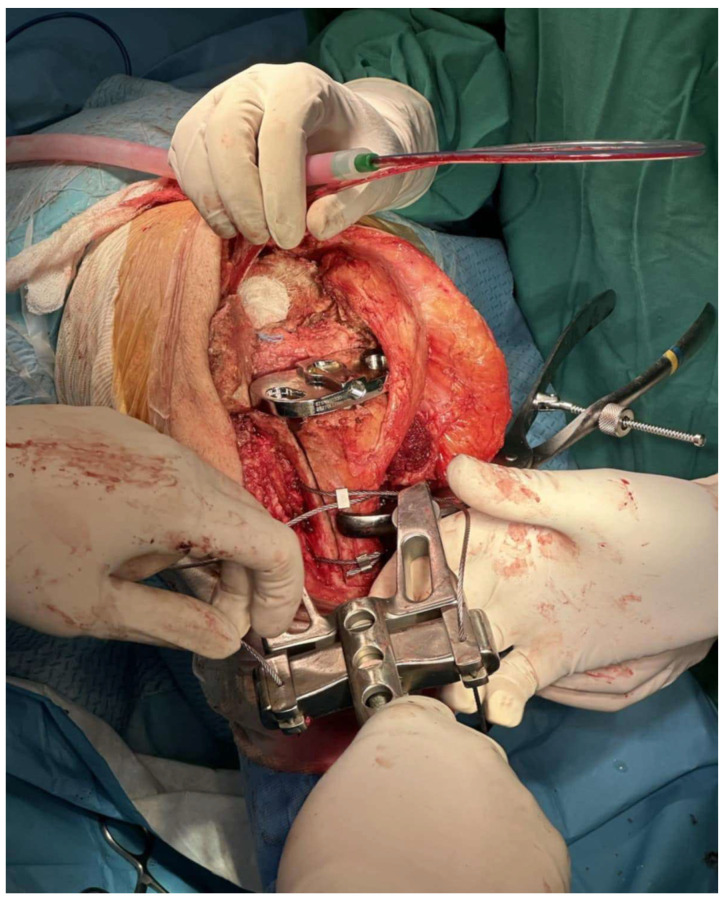
Cerclage wires reducing the bone flap with the final tibia implant in place. Note the length and transverse cut of the osteotomy (Case 3).

Preparation of the intramedullary canal followed, and trial implants were then tested. The final implants were considered as “hybrid”, since the metaphyseal part was fixed with cement, whereas the stem was uncemented. In cases where tibial reconstruction was needed because of the osteotomy, we typically proceeded with the implantation in two (2) stages, one for the femoral component and one for the tibial one. For the tibia (second stage), the bone fragment was reduced manually with the final tibial implant in place and the knee in a 90-degree flexion. Usually, there was no need for reduction forceps to obtain reduction. One should be very cautious when reduction forceps are used, to avoid fragmentation of the bone flap as it is very thin and fragile. Obtaining a near-anatomic reduction was crucial for osteotomy union. Three wire cerclages were passed to stabilize the bone fragment using a cerclage wire passer (Figure 2). The distance between them depended on the osteotomy length. The wires were tightened sequentially on the medial side of the tibia, starting from the most distal and proceeding to the most proximal.

**Figure 3:**
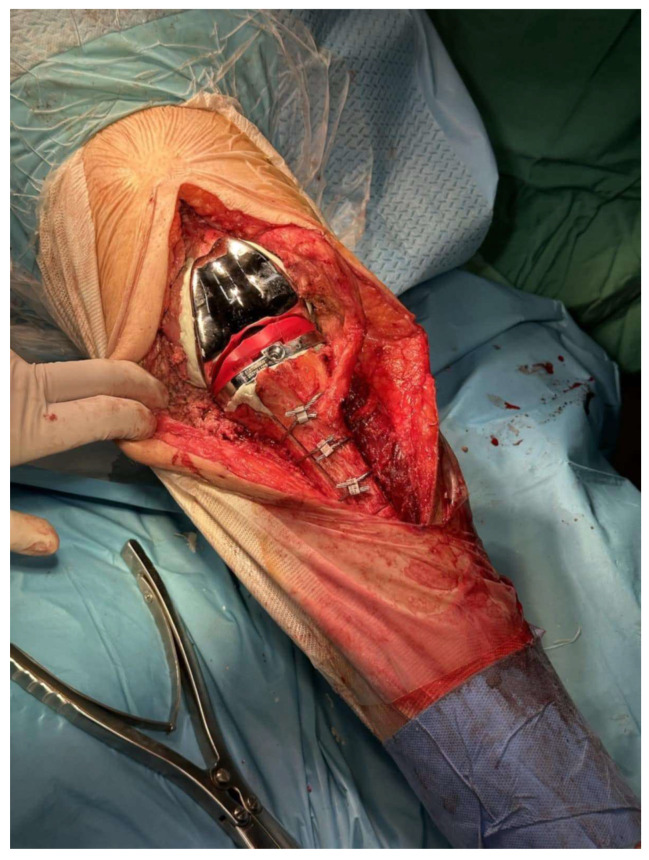
Final construct with three cerclage wires and final implants. Cement excess is removed from the osteotomy site (Case 3).

**Figure 4:**
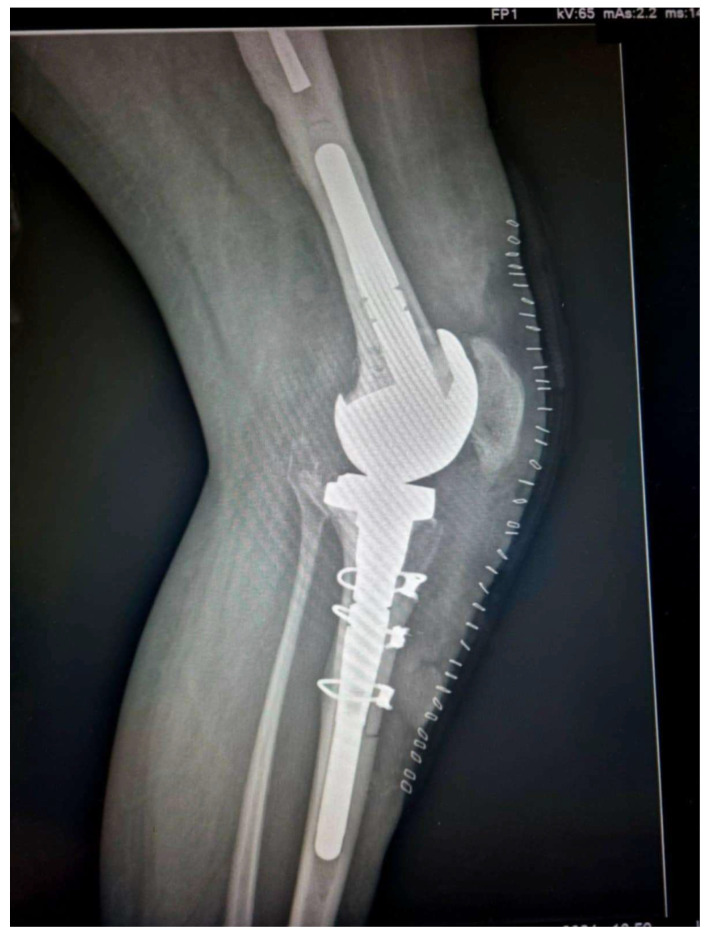
Postoperative radiograph demonstrating the proper reduction of the bone flap (Case 3).

Caution must be given when passing behind the posterior tibia so as not to traumatize the neurovascular bundle. The passer must cross as close as possible to the posterior cortex of the tibia. Afterwards, the tibial component was removed and re-implanted, with bone cement applied solely to the central-metaphyseal region (Figure 3 and Figure 4). Any cement excess was removed from the osteotomy site.

Finally, patellofemoral tracking was inspected, and the patella was resurfaced. Lateral retinaculum release was performed when indicated. The tourniquet was deflated, and a thorough hemostasis was completed. The entire synovium and posterior capsule were infiltrated with a mixture of normal saline with epinephrine, rovipacaine, and tranexamic acid to reduce bleeding. A suction drain was placed, and medial parapatellar arthrotomy was closed with interrupted sutures.

All patients wore compression stockings, whereas no antithrombotic prophylaxis was administered. Intravenous vancomycin was administered 30 min before incision and was continued for another 48 h after surgery. The suction drain was removed on the 2nd day after surgery, and the factor levels were monitored daily. Hospitalization routinely lasted 5–7 days.

Postoperatively, a knee immobilizer in full extension was applied for the first 72 h. Knee flexion was then permitted up to 90 degrees for the initial four weeks, followed by gradual progression to full range of motion thereafter. Mobilization began on the first postoperative day with partial weight bearing using crutches or a walker. Full weight bearing was typically allowed at six weeks postoperatively.

## 3. Results

**Figure 5:**
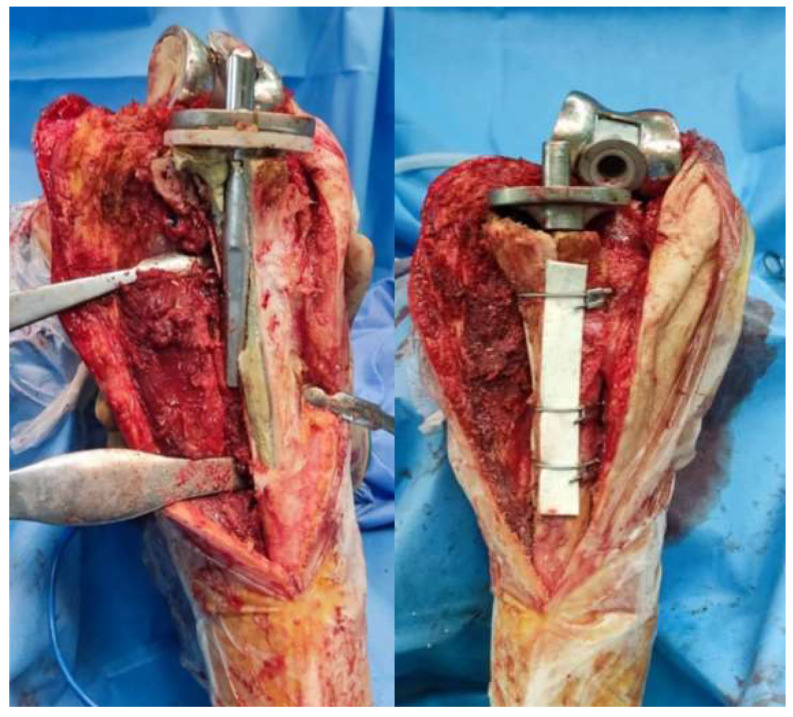
Intraoperative photograph after extended tibial tuberosity osteotomy demonstrating tibial stem malposition with cortical perforation. Final construct after osteotomy reduction with the use of three cerclage wires and bone graft (Case 5).

We retrospectively reviewed seven (7) hemophilic patients, with a mean age of 57.5 ± 1.50 years (95% confidence interval: 56–60 years) and mean body mass index (BMI) of 26.07 ± 0.67 kg/m^2^ (95% confidence interval: 25–27 kg/m^2^), who underwent ETTO for revision TKA in our department between 2015 to 2023. Preoperative diagnosis included (a) aseptic loosening of the femoral component but with a well-fixed tibial stem in four cases and (b) tibial component malposition that caused marked axial deformity in three cases (Figure 5). All seven patients initially considered for the study were included in the final analysis. None met the exclusion criterion—namely, the presence of active infection—at the time of evaluation, and all subsequently underwent surgery. The same surgical technique was applied in all seven cases. In Case 5 (Figure 5), the ETTO fragment was augmented with a bone graft prior to final reduction using cerclage wires, due to a bone defect attributed to prior stem perforation. A rotating hinge prosthesis was implanted in all patients, with an uncemented long stem. Clinical and radiological evaluation was performed at 6 weeks and 3, 6, and 12 months after surgery. Full baseline characteristics and outcomes are described in Table 1, Table 2 and Table 3.

**Figure 6:**
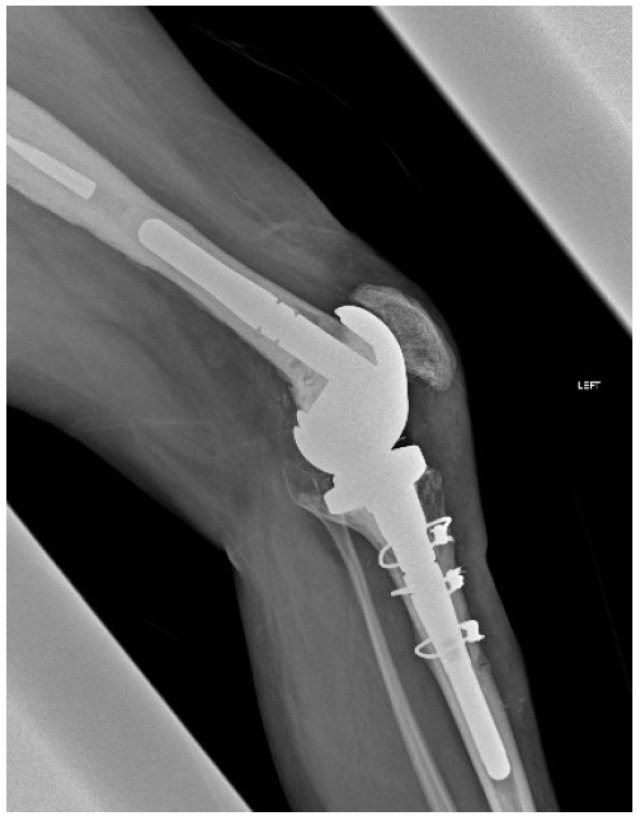
Lateral radiograph demonstrating bridging callus formation at the osteotomy site (Case 3).

All patients showed union at the osteotomy site 4 months after surgery, as confirmed by the presence of bridging callus formation on the lateral radiograph (Figure 6). Furthermore, there were no major complications related to the ETTO, such as infection, stem perforation or loosening, wire breakage, or periprosthetic fracture. There was only one case of delayed wound healing of the skin incision, probably attributed to the history of smoking and the presence of an inhibitor, which was managed with surgical debridement and antibiotic administration. No extension lag was recorded, and mean postoperative knee flexion was noted at 92.0° ± 2.58° (95% confidence interval: 8°–95°). In all cases, ETTO was proven to be a mandatory tool for performing a successful total knee revision.

## 4. Discussion

To our knowledge, this is the first study to specifically evaluate the application of an ETTO in a cohort of hemophilic patients undergoing revision total knee arthroplasty. While ETTO has been previously described in complex revision cases, its adaptation in hemophilia—a population with fragile bone structure and elevated bleeding risk—represents a novel and clinically significant extension of the technique. This study highlights not only its technical feasibility but also its safety and effectiveness in this high-risk subgroup.

Well-fixed long tibial stems, cemented or cementless, and difficult knee exposure due to patella baja, arthrofibrosis, or marked stiffness of the knee often necessitate the use of a TTO. This is a practical technique, first described by Dolin [7] and then modified by Whiteside and Ohl. Many variations have been reported ever since. Extended TTO can be successfully performed even in cases of previous TTO or following two-stage revision due to infection [8]. A relative contraindication for ETTO is a massive tibial osteolysis that could compromise the fragment fixation [9,10]. ETTO demonstrates good results with high union and low complication rates, representing an efficient and safe choice for the orthopedic surgeon in certain indications. Fragment displacement, nonunion, extensor lag due to proximal fragment migration, symptomatic hardware, and delayed wound healing over the osteotomy site are also reported as possible complications following an ETTO.

Whiteside demonstrated excellent results after a total of 136 TKA cases utilizing TTO [6]. Range of motion following surgery was a mean of 93 degrees. There were two cases of residual extensor lag, two cases of fracturing of the osteotomy fragment with some proximal migration, and three cases of symptomatic hardware that required removal. However, all osteotomies united, including those performed in infected cases. There were also three tibial fractures following surgery.

Mendes et al. reported on a large retrospective study of revision TKAs in 67 knees where a step cut TTO was used for exposure [11]. If the proximal tibial bone stock was insufficient to perform a step cut, the fragment was reduced against the anterior edge of the tibial component. The results were good to excellent in 87% of cases, with a mean KSS of 86 points. The mean postoperative flexion was 107 degrees. There were 11 patients who complained of tenderness when palpated on the wire site, and 2 of them required removal of the wires. Symptomatic nonunion developed in two cases, and two patients presented extensor lag due to proximal migration of the osteotomy fragment. There was only one tibial fracture that occurred during closed manipulation, performed for the treatment of postoperative stiffness.

Cance et al. reviewed 135 patients who received an RTKA with concomitant TTO [12]. They reported healing of the osteotomy in 128 patients (95%). There were nine cases of fracture displacement of the TTO and five cases of aseptic nonunion. No postoperative extension lag was noted.

Van den Broek et al. reviewed the use of TTO in 39 cases of revision TKA [13]. They demonstrated successful osteotomy healing in 37 of 39 patients. Their experience revealed two cases of proximal translation. There were no cases of extensor lag.

Byron E Chalidis and Michael D Ries evaluated 87 TTOs performed during RTKAs with an average follow-up of 49 months [8]. Osteotomy healed in all cases. Avulsion of the proximal part of the tibial tubercle was noted in three patients, and proximal migration of the bone fragment in another two cases. Skin necrosis developed in one of the knees with proximal migration requiring a medial gastrocnemius muscle flap transposition. This patient had a history of rheumatoid arthritis and steroid use.

Postoperative stiffness can be managed with closed manipulation under anesthesia [8,11]. Great caution must be given due to the high risk of iatrogenic fracture. Fragment displacement in cases of unaccepted position and presence of extensor lag can be treated with reduction osteosynthesis, and nonunion, a rare complication as indicated by the existing literature, with open reduction and bone grafting [12,13]. Symptomatic hardware (screws or wires) can be removed once the osteotomy site heals [6,8,9,10,13].

Bicortical screws and cerclage wires are the two common techniques for osteotomy fixation. Small screws are preferred because they reduce the risk of fracturing the fragment of the TTO [13]. Davis and Caldwell et al. found a biomechanical advantage of screw fixation versus wire fixation when experimenting with fresh-frozen specimens [14]. The strongest fixation is obtained by two bicortical 4.5 mm screws slightly ascendant [12]. Cerclage wires constitute a safe alternative resulting in a very stable fixation, avoiding the stress-rising effect of drill holes for screw fixation. This option is optimal in cases of low-thickness bone flaps and when screws cannot be placed due to the presence of a large metaphyseal cone or sleeve [12]. However, wire fixation can theoretically cause proximal fragment migration [15].

While most of the existing literature on tibial tuberosity osteotomy techniques focuses on non-hemophilic populations, our study extends these findings into a highly specific and surgically challenging cohort. Hemophilic patients present unique risks, including compromised bone quality and increased bleeding tendencies, which significantly elevate the complexity of revision arthroplasty. Despite these challenges, our results demonstrate outcomes comparable to those reported in broader populations regarding osteotomy union rates, complication profiles, and knee function recovery. This reinforces the value of ETTO not only as a viable technique but as a particularly suitable method in hemophilic patients requiring complex implant removal during revision TKA. Our findings align with previously published data from large revision series in non-hemophilic populations, while highlighting the feasibility and safety of this approach in hemophilic patients—a group underrepresented in the orthopedic revision literature.

Unlike conventional TTO, which often employs a shorter, proximal step cut and tapered distal configuration to facilitate patellar eversion and exposure, our ETTO technique utilizes two long, parallel sagittal cuts along the anterior tibial shaft, connected distally by a straight transverse cut, resulting in a monoblock osteotomy fragment that includes the tibial tubercle. This longer and linear design provides extended exposure of the intramedullary canal, enabling safe extraction of well-fixed or cemented tibial stems without compromising the remaining bone stock.

In our technique (Graphical Abstract), we reduce the bone fragment manually, with the final tibial component provisionally in place. If the fragment does not reduce anatomically, we carve the undersurface of the bone fragment with a rongeur or an oscillating saw until a perfect fit is acquired. Afterwards, we pass and tighten the three wires circumferentially, without any drill holes in the tibia, remove the tibial stem, and reimplant it with bone cement applied solely to the central-metaphyseal region. We always check for stability of fragment fixation in a wide range of motion of the knee joint. This technique allows for a near-perfect reduction of the bone fragment in order to achieve the best union results. Any interfering cement in the osteotomy site is also removed for the same purpose. In comparison with other techniques, we do not perform a proximal step cut, and the osteotomy is extended in the knee articular surface. The anterior border of the tibial baseplate acts as a buttress in ETTO proximal migration. In our case series, we did not report any fragment displacement or nonunion. Tibial component rotation is calculated intraoperatively using the center of the ankle joint and the second metatarsal as landmarks, and the tibial stem must of course bypass the distal end of the osteotomy. Furthermore, we retain as much soft tissue in the lateral tibia as possible. We detach only the bare minimum of lateral muscles in order to perform the lateral tibia parallel cut. All these key steps aim at an anatomical and stable reduction of the bone fragment and vascularization preservation, achieving a high union rate.

### Limitations

This study has several limitations that should be considered when interpreting the results. First, the sample size is small (*n* = 7), which limits the statistical power and generalizability of the findings. However, this reflects the inherent rarity of hemophilic patients requiring revision TKA, particularly those necessitating ETTO. Even in our institution—a referral center with dedicated expertise in hemophilia management—such complex cases accumulate slowly over time. Second, the study was retrospective in nature, and while detailed operative data and postoperative outcomes were collected, standardized functional outcome scores were not uniformly available due to inconsistent documentation in the early part of the study period. Third, although all patients were followed for 12 months, long-term outcomes remain unknown. An extended follow-up would provide a more comprehensive evaluation of technique durability. Fourth, the surgical technique was performed by a single senior surgeon experienced in complex revision arthroplasty. The senior author and operating surgeon has performed over 35 ETTO procedures over the past 13 years, involving both hemophilic and non-hemophilic patients. While this ensures consistency, it may also limit external reproducibility, especially in centers without similar expertise in both hemophilia care and complex knee revision. Finally, although descriptive statistics and confidence intervals were provided, the small cohort size precluded more advanced statistical analyses or hypothesis testing. Future prospective studies across multiple centers may help validate these findings and explore predictors of outcomes in this unique patient population.

## 5. Conclusions

Patients with severe hemophilic arthropathy of the knee typically undergo a TKA at a young age, and for many reasons, they show a higher risk for revision. ETTO is a complex yet very useful technique allowing for safe tibial stem extraction during difficult TKA revisions. This technique demonstrates good functional results with high union and low complication rates. However, due to its complexity, it should be performed only by experienced surgeons in certain referral centers.

## Figures and Tables

**Table 1:** Baseline characteristics of patients undergoing extended tibial tuberosity osteotomy.

Patient ID	Age (Years)	Gender	BMI (kg/m^2^)	Side (L/R)	Preop Diagnosis	Implant Type
Case 1	56	Male	25.5	R	Aseptic loosening	Rotating hinge
Case 2	58	Male	26.0	L	Aseptic loosening	Rotating hinge
Case 3	59	Male	26.5	L	Malposition	Rotating hinge
Case 4	57	Male	26.0	L	Aseptic loosening	Rotating hinge
Case 5	58	Male	25.0	R	Malposition	Rotating hinge
Case 6	56	Male	26.5	L	Malposition	Rotating hinge
Case 7	60	Male	27.0	R	Aseptic loosening	Rotating hinge

**Table 2:** Patients’ comorbidities.

Patient ID	Osteoporosis	Diabetes Mellitus	Smoking History	Previous Tibial Osteotomy
Case 1	Yes	No	Yes	No
Case 2	No	Yes	No	No
Case 3	No	No	No	No
Case 4	Yes	No	Yes	No
Case 5	No	No	No	No
Case 6	No	Yes	No	No
Case 7	Yes	No	Yes	No

**Table 3:** Postoperative outcomes of extended tibial tuberosity osteotomy patients.

Patient ID	Osteotomy Union	Complications	Extension Lag	Postop Knee Flexion (°)	Maximum Follow-Up Duration (Months)
Case 1	Yes	None	No	90	12
Case 2	Yes	None	No	95	12
Case 3	Yes	None	No	88	12
Case 4	Yes	Delayed wound healing	No	92	12
Case 5	Yes	None	No	93	12
Case 6	Yes	None	No	91	12
Case 7	Yes	None	No	95	12

## Data Availability

The raw data supporting the conclusions of this article will be made available by the authors on request.
